# Identification of a Susceptibility Locus for Severe Adolescent Idiopathic Scoliosis on Chromosome 17q24.3

**DOI:** 10.1371/journal.pone.0072802

**Published:** 2013-09-04

**Authors:** Atsushi Miyake, Ikuyo Kou, Yohei Takahashi, Todd A. Johnson, Yoji Ogura, Jin Dai, Xusheng Qiu, Atsushi Takahashi, Hua Jiang, Huang Yan, Katsuki Kono, Noriaki Kawakami, Koki Uno, Manabu Ito, Shohei Minami, Haruhisa Yanagida, Hiroshi Taneichi, Naoya Hosono, Taichi Tsuji, Teppei Suzuki, Hideki Sudo, Toshiaki Kotani, Ikuho Yonezawa, Michiaki Kubo, Tatsuhiko Tsunoda, Kota Watanabe, Kazuhiro Chiba, Yoshiaki Toyama, Yong Qiu, Morio Matsumoto, Shiro Ikegawa

**Affiliations:** 1 Laboratory for Bone and Joint Diseases, RIKEN Center for Integrative Medical Science, Tokyo, Japan; 2 Department of Orthopaedic Surgery, School of Medicine, Keio University, Tokyo, Japan; 3 Laboratory for Medical Science Mathematics, RIKEN Center for Integrative Medical Science, Yokohama, Japan; 4 Department of Orthopaedics, The Center of Diagnosis and Treatment for Joint Disease, Drum Tower Hospital Affiliated to Medical School of Nanjing University, Nanjing, China; 5 Department of Spine Surgery, Drum Tower Hospital Affiliated to Medical School of Nanjing University, Nanjing, China; 6 Laboratory for Statistical Analysis, RIKEN Center for Integrative Medical Science, Tokyo, Japan; 7 Scoliosis Center, Saiseikai Central Hospital, Tokyo, Japan; 8 Department of Orthopaedic Surgery, Meijo Hospital, Nagoya, Japan; 9 Department of Orthopaedic Surgery, National Hospital Organization, Kobe Medical Center, Kobe, Japan; 10 Department of Advanced Medicine for Spine and Spinal Cord Disorders, Hokkaido University Graduate School of Medicine, Sapporo, Japan; 11 Department of Orthopaedic Surgery, Seirei Sakura Citizen Hospital, Sakura, Japan; 12 Department of Orthopaedic Surgery, Fukuoka Children's Hospital, Fukuoka, Japan; 13 Department of Orthopaedic Surgery, Dokkyo Medical University School of Medicine, Mibu, Japan; 14 Laboratory for Genotyping Development, RIKEN Center for Integrative Medical Science, Yokohama, Japan; 15 Department of Orthopaedic Surgery, Juntendo University School of Medicine, Tokyo, Japan; 16 Department of Orthopaedic Surgery, Kitasato University Kitasato Institute Hospital, Tokyo, Japan; The Children's Hospital of Philadelphia, United States of America

## Abstract

Adolescent idiopathic scoliosis (AIS) is the most common spinal deformity, affecting around 2% of adolescents worldwide. Genetic factors play an important role in its etiology. Using a genome-wide association study (GWAS), we recently identified novel AIS susceptibility loci on chromosomes 10q24.31 and 6q24.1. To identify more AIS susceptibility loci relating to its severity and progression, we performed GWAS by limiting the case subjects to those with severe AIS. Through a two-stage association study using a total of ∼12,000 Japanese subjects, we identified a common variant, rs12946942 that showed a significant association with severe AIS in the recessive model (*P* = 4.00×10^−8^, odds ratio [OR] = 2.05). Its association was replicated in a Chinese population (combined *P* = 6.43×10^−12^, OR = 2.21). rs12946942 is on chromosome 17q24.3 near the genes *SOX9* and *KCNJ2*, which when mutated cause scoliosis phenotypes. Our findings will offer new insight into the etiology and progression of AIS.

## Introduction

Adolescent idiopathic scoliosis (AIS) is the most common structural deformity of the spine, occurring in 2–3% of healthy children from the age of 10 to skeletal maturity worldwide [1]. A Japanese study showed that the incidence of scoliosis of more than 15 degrees increases linearly with age from 10 (0.07% in boys, 0.44% in girls) to 14 (0.25% in boys, 1.77% in girls), and that most of these cases are AIS [2].

AIS is a multi-factorial disorder, with genetic factors playing an important role in its etiology [3]. Population studies have shown that its familial incidence is higher than that in general populations [4], while twin studies have consistently shown higher concordance in monozygotic compared with dizygotic twins. For example, a meta-analysis of several twin studies revealed 73% monozygotic and 36% dizygotic twin concordance [5]. Using the Danish Twin Registry, Andersen et al. observed 25% proband-wise concordance in monozygotic twins (six of 44 concordant) compared with 0% concordance in dizygotic twins (0 of 91), with an overall prevalence of approximately 1% [6].

Several genetic studies regarding AIS susceptibility have previously been reported. Although genome-wide linkage analyses have revealed some AIS susceptibility loci [7]–[16], only *CHD7* has been identified as a susceptibility gene [13]. Genetic association studies of AIS, however, have identified several predisposition genes. Single nucleotide polymorphisms (SNPs) in *ESR1*, *ESR2*, *MATN1*, *MTNR1B*, and *TPH1* genes are reported to be associated with AIS susceptibility [17]–[21]. Recently, we used genome-wide association study (GWAS) to identify novel AIS susceptibility locus on chromosomes 10q24.31 near the *LBX1* gene [22] and 6q24.1 in the *GPR126* gene [23].

We used a common-control design [24], [25] in our previous GWAS. However, because undiagnosed general populations or patients with unrelated diseases are used as controls in this design, there is a potential loss of power associated with the inability to exclude latent diagnoses of the disease. One way of overcoming this is to adopt a more stringent case definition; for example, one based on early age of onset or the identification of a more severe disease phenotype [26]. Severe cases are presumed to have a high prevalence of susceptibility alleles for that disease and lower phenotypic heterogeneity, and will hence improve the study power by enriching for specific disease-predisposing alleles. In addition, it is clinically important to consider the factors that influence the progression of scoliosis, as the treatment of AIS patients depends on its severity and possibility of progression. Both of these factors have a genetic component [27], [28]: the Danish meta-analysis twin study showed a significant correlation with curve severity in monozygous but not dizygous twins [5], while SNPs in *ESR1*, *ESR2*, *MATN1* and *IGF1* genes are associated with AIS severity [17]–[19], [29]. Studies into severe AIS may clarify the factors that influence AIS progression.

In the current study, we performed GWAS by including in our case group only severely affected AIS subjects with a Cobb's angle above 40°. We have identified a novel susceptibility locus for severe AIS on chromosome 17q24.3 that showed genome-wide significance and replication of association in different ethnic populations.

## Materials and Methods

### Subjects

We defined Cobb's angle for severe AIS was above 40°. Cobb's angles were obtained at the time the patient was recruited in this study. A written informed consent was obtained from all subjects participating in the study. The study was approved by the institutional review boards of RIKEN and participating institutions. The subjects for the GWAS were all Japanese females; 554 with severe AIS (aged 10–39) and 1,474 control subjects (aged 7–96) were recruited as previously described [22]. For the Japanese replication study, we recruited an independent set of a case-control subjects consisting of 268 severe AIS (aged 10–59) and 9,823 controls (aged 20–96) in the same way. For examining the relation between the genotype of the SNPs identified by the case-control association study and the AIS severity (Cobb angle) in Japanese, we collected the data of 1,767 AIS subjects used for the previous GWAS and replication studies who had AIS with a Cobb angle of 10° or greater. All were Japanese female. For the replication study in Chinese, we recruited 571 females with severe AIS (aged 10–19) and 326 female controls (aged 25–83) living in and around Nanjing city, China. All were self-reported Han Chinese.

### Genotyping of SNPs and Quality Control

Genomic DNA was extracted from the peripheral blood leukocytes of severe AIS and control subjects using standard protocols. In the GWAS, we genotyped case subjects using the Illumina Human610 Genotyping BeadChip and control subjects with the Illumina HumanHap550v3 Genotyping BeadChip. SNPs common to both platforms were then combined and analyzed as previously described [30]. Inadequate SNPs and subjects were checked and excluded as previously described [22]. In the Japanese replication study, the case subjects were genotyped using the PCR-based Invader assay [31] and controls were genotyped using Illumina HumanHap550v3 Genotyping BeadChip. In the Chinese replication study, all subjects were genotyped using the PCR-based Invader assay as described above.

### Statistical Analysis

The association between the SNPs was examined by *x* ^2^ test for three models (allele model, recessive model and dominant model) and minimum *P* values in the three models were evaluated. In the same way, incidence of severe AIS, and the Hardy–Weinberg equilibrium (HWE) of the genotypes were examined by *x* ^2^ test. Data of GWAS and Japanese replication study were combined by addition, and data of total Japanese studies and Chinese study were combined using the Mantel-Haenszel method. The Breslow-Day statistic was used to test homogeneity of the common odds ratio. The associations between the SNP genotypes and the Cobb angle of AIS subjects were evaluated using the Kruskal-Wallis and Mann-Whitney U tests. Imputation was performed using MACH version 1.0.16.c. and Minimac software with reference haplotypes from the 1000 Genomes Project June 2011 EAS population as described elsewhere [23]. In the analysis, pairwise *r* ^2^ values were calculated using the R Bioconductor package snpMatrix (version 1.16.2), and the LD map was created using in-house programs. We performed association analysis of imputed data using the single.snp.tests function in the R package snpStats version 1.3.4 after converting Minimac output to the uncertain genotype data format for snpStats. Regional association plots were generated using R statistical environment version 2.13.0.

## Results

After stringent quality control of the subjects and SNPs, we examined the association of 455,121 SNPs with severe AIS using the χ^2^ test for three models (allele model, recessive model and dominant model). No SNP reached the GWAS significance threshold (*P*<5×10^−8^) at this stage (**Figure S1**).

Then, we selected 27 SNPs (**Table S1**) according to the following criteria: 1) a minimum *P* value in the three models <1×10^−4^; 2) a minor allele frequency ≥0.1. SNPs in strong linkage disequilibrium (LD) with a correlation coefficient (*r* ^2^) of 0.8 with other SNPs were excluded from analysis. We checked their association using an independent set of Japanese female case-control subjects and combined all Japanese data.

Six SNPs showed association of genome-wide significance level (P<5×10^−8^) (**Table 1**). Five of them were in the known loci of AIS susceptibility that we previously reported; three SNPs (rs11190870, rs625039 and rs11598564) were close to *LBX1* on chromosome 10q24.31 [22] and two SNPs (rs6570507 and rs9496346) were on chromosome 6q24.1 in the *GPR126* gene [23]. In addition, rs12946942 on chromosome 17q24.3 showed significant association in the recessive model (*P* = 4.00×10^−8^, odds ratio [OR]  = 2.05). We further examined the relation between the rs12946942 genotypes and the AIS severity (Cobb's angle) using a total of 1,767 AIS cases. rs12946942 showed significant association (*P* = 3.02×10^−2^; by the Kruskal-Wallis test).

**Table 1:** Association of the 27 SNPs in the two-stage association study for AIS in Japanese.

dbSNP ID	Chromosome	RAF		*P* value^a^			Odds ratio (95% CI)		
		case	contrl	allele	recessive	dominant	allele	recessive	dominant
rs11190870	10q24.31	0.675	0.565	**2.80×10^−18^**	**1.57×10^−13^**	**9.99×10^−12^**	1.60 (1.44–1.78)	1.71 (1.48–1.97)	2.24 (1.76–2.84)
rs625039	10q24.31	0.734	0.636	**1.28×10^−15^**	**1.29×10^−12^**	**6.14×10^−9^**	1.58 (1.41–1.77)	1.67 (1.44–1.92)	2.32 (1.73–3.10)
rs925203	5p15.31	0.446	0.398	1.46×10^−4^	5.28×10^−4^	4.85×10^−3^	1.22 (1.10–1.34)	1.36 (1.14–1.63)	1.24 (1.07–1.45)
rs7545121	1q31.1	0.590	0.551	2.07×10^−3^	1.78×10^−2^	6.72×10^−3^	1.17 (1.06–1.30)	1.20 (1.03–1.39)	1.30 (1.08–1.58)
rs12946942	17q24.3	0.258	0.211	9.58×10^−6^	**4.00×10^−8^**	3.57×10^−3^	1.30 (1.16–1.45)	2.05 (1.58–2.66)	1.24 (1.07–1.43)
rs11598564	10q24.31	0.547	0.460	**9.77×10^−12^**	1.02×10^−7^	**9.03×10^−9^**	1.42 (1.28–1.57)	1.53 (1.31–1.79)	1.67 (1.40–1.99)
rs267766	5p13.2	0.297	0.246	4.30×10^−6^	8.38×10^−3^	1.27×10^−5^	1.29 (1.16–1.44)	1.41 (1.09–1.82)	1.37 (1.19–1.58)
rs1367272	2p25.1	0.594	0.547	1.77×10^−4^	2.43×10^−4^	1.59×10^−2^	1.22 (1.10–1.35)	1.32 (1.14–1.53)	1.26 (1.04–1.53)
rs2676801	17q21.33	0.758	0.722	1.41×10^−3^	6.74×10^−4^	2.38×10^−1^	1.21 (1.08–1.36)	1.28 (1.11–1.48)	1.18 (0.90–1.56)
rs2047176	5p13.2	0.461	0.401	1.95×10^−6^	7.02×10^−5^	1.31×10^−4^	1.28 (1.15–1.41)	1.42 (1.19–1.69)	1.35 (1.16–1.58)
rs6570507	6q24.1	0.499	0.430	**3.78×10^−8^**	1.70×10^−4^	3.66×10^−7^	1.32 (1.20–1.46)	1.38 (1.16–1.62)	1.53 (1.30–1.80)
rs655540	11q24.2	0.376	0.332	2.61×10^−4^	4.34×10^−5^	2.05×10^−2^	1.21 (1.09–1.35)	1.51 (1.24–1.84)	1.19 (1.03–1.37)
rs9405284	6p25.1	0.738	0.690	5.85×10^−5^	3.21×10^−5^	5.93×10^−2^	1.26 (1.13–1.41)	1.35 (1.17–1.56)	1.30 (0.99–1.70)
rs7895098	10q23.1	0.888	0.851	5.04×10^−5^	6.96×10^−5^	7.84×10^−2^	1.38 (1.18–1.62)	1.42 (1.19–1.69)	1.71 (0.93–3.15)
rs9496346	6q24.1	0.510	0.438	**1.00×10^−8^**	4.72×10^−5^	2.26×10^−7^	1.34 (1.21–1.48)	1.40 (1.19–1.65)	1.55 (1.31–1.83)
rs346981	1p22.2	0.532	0.497	5.88×10^−3^	1.77×10^−2^	3.46×10^−2^	1.15 (1.04–1.27)	1.21 (1.03–1.42)	1.20 (1.01–1.42)
rs2852199	11q22.3	0.325	0.295	1.01×10^−2^	5.24×10^−2^	2.58×10^−2^	1.15 (1.03–1.28)	1.26 (1.00–1.59)	1.18 (1.02–1.36)
rs4076823	16p13.13	0.647	0.613	7.39×10^−3^	3.73×10^−2^	1.89×10^−2^	1.15 (1.04–1.28)	1.17 (1.01–1.35)	1.30 (1.04–1.61)
rs7101916	11q13.1	0.490	0.454	4.59×10^−3^	1.46×10^−2^	2.56×10^−2^	1.16 (1.05–1.28)	1.23 (1.04–1.45)	1.20 (1.02–1.40)
rs10485749	20p12.2	0.693	0.647	1.65×10^−4^	9.55×10^−4^	5.49×10^−3^	1.23 (1.10–1.37)	1.27 (1.10–1.46)	1.41 (1.11–1.80)
rs11227247	11q13.1	0.482	0.447	5.98×10^−3^	1.78×10^−2^	3.30×10^−2^	1.15 (1.04–1.27)	1.22 (1.03–1.45)	1.19 (1.01–1.39)
rs12346254	9p24.1	0.761	0.724	1.07×10^−3^	2.73×10^−3^	3.38×10^−2^	1.22 (1.08–1.37)	1.25 (1.08–1.44)	1.38 (1.02–1.86)
rs10485285	6q14.1	0.565	0.521	5.48×10^−4^	3.45×10^−3^	7.30×10^−3^	1.19 (1.08–1.32)	1.25 (1.08–1.46)	1.28 (1.07–1.53)
rs9918553	7p14.1	0.743	0.706	1.54×10^−3^	3.07×10^−2^	6.03×10^−4^	1.20 (1.07–1.35)	1.17 (1.01–1.35)	1.72 (1.26–2.36)
rs7143583	14q21.2	0.914	0.888	1.37×10^−3^	1.38×10^−3^	2.85×10^−1^	1.34 (1.12–1.59)	1.36 (1.13–1.65)	1.51 (0.71–3.24)
rs17012036	4q28.1	0.259	0.218	1.13×10^−4^	1.13×10^−1^	8.45×10^−5^	1.25 (1.12–1.41)	1.27 (0.94–1.71)	1.33 (1.15–1.53)
rs454578	5q14.1	0.270	0.239	4.19×10^−3^	5.13×10^−2^	1.01×10^−2^	1.18 (1.05–1.32)	1.31 (1.00–1.71)	1.20 (1.05–1.39)

Combined results of GWAS and the replication study. RAF: risk allele frequency. CI: confidence interval.

a calculated by *x* ^2^ test. *P* values below the genome-wide significance level (P<5×10^−8^) are in bold.

We performed a replication study for rs12946942 in a Chinese case-control population. The association of rs12946942 was significant in the Chinese population for all three models. Combined *P* values from the Mantel-Haenszel method for the Japanese and Chinese studies in the recessive model showed genome-wide significance (*P* = 6.43×10^−12^) (**Table 2**).

**Table 2:** Association of rs12946942 with severe AIS in Japanese and Chinese populations.

Population	Study	RAF		*P* value^a^	Odds ratio	*P*_BD_ b
		case	control		(95% CI)	
Japanese	GWAS	0.274	0.203	1.95×10^−5^	2.24 (1.53–3.27)	
	Replication	0.224	0.213	6.09×10^−2^	1.59 (0.97–2.59)	
	Total	0.258	0.211	4.00×10^−8^	2.05 (1.58–2.66)	
Chinese	Replication	0.392	0.288	3.27×10^−5^	2.59 (1.63–4.10)	
Combined	Meta- analysis^c^			6.43×10^−12^	2.21 (1.76–2.77)	0.38

RAF: risk allele (T allele) frequency. CI: confidence interval.

Data of *P* value, odds ratio (95% CI) and P_BD_ are for the recessive model (G/G and G/T vs T/T).

a by *x* ^2^ test.

b homogeneity of odds ratios by the Breslow-Day test.

c by the Mantel-Haenszel method.

**Figure 1:**
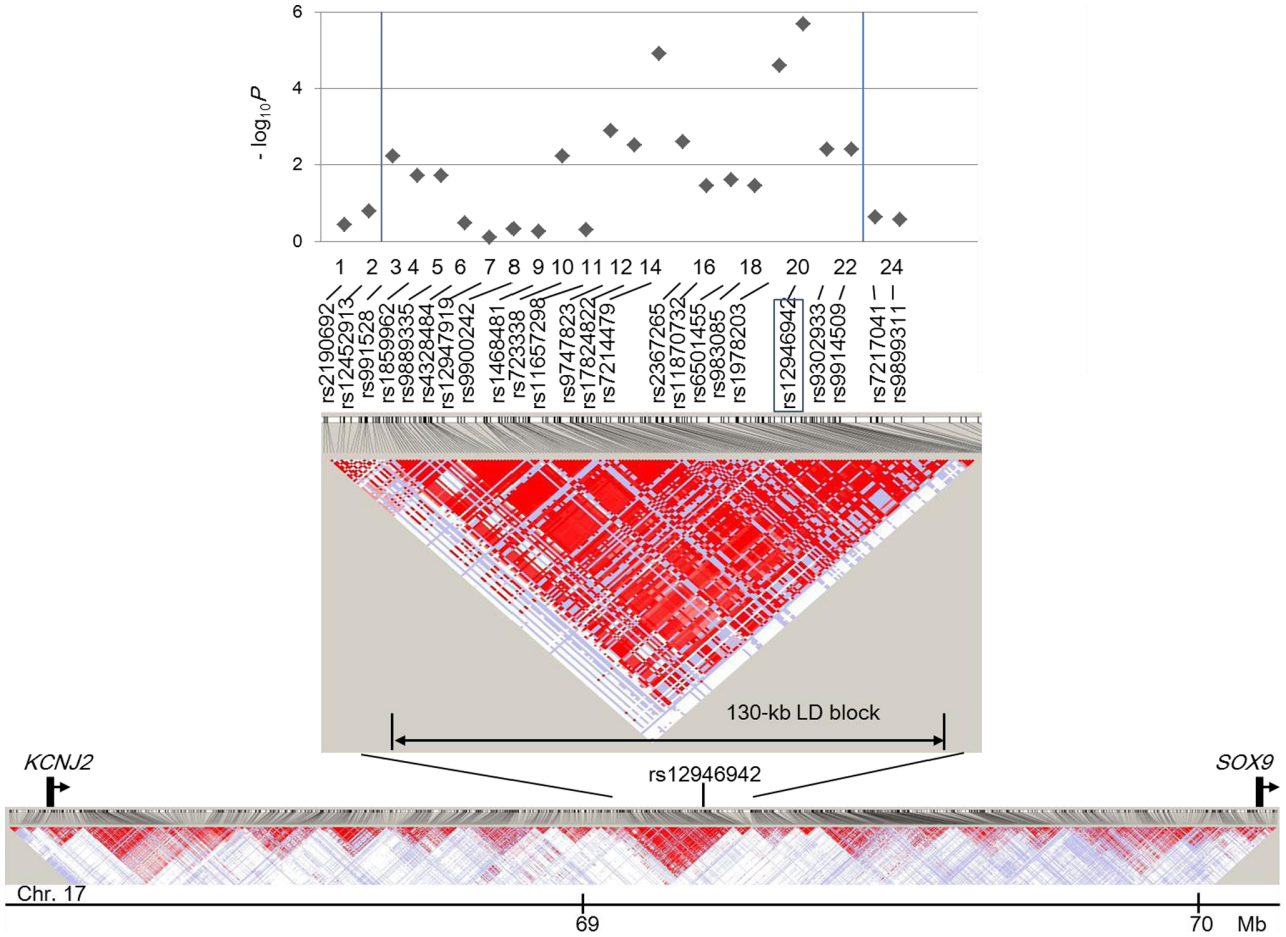
Linkage disequilibrium (LD) map and *P*-value plot of the severe adolescent idiopathic scoliosis susceptibility locus at chromosome 17q24.3. Top panel: The association results shown as – log_10_ of minimum *P* values in allele, recessive and dominant models and a focus view of the 130-kb LD block including rs12946942. All SNPs are analyzed in GWAS. Two vertical lines in this graph indicate the range of the LD block. rs12946942 is boxed. Middle panel: The ∼2 Mb LD map (D') around rs12946942 is shown using loci with MAF >0.10 from Phase II HapMap (release 24) JPT individuals. LD score: (dark red) LOD >2, D' = 1; (light red) LOD >2, D'<1; (blue) LOD <2, D' = 1; (white) LOD <2, D'<1. Bottom panel: The position of rs12946942 and the two candidate genes (*KCNJ2* and *SOX9*) on chromosome (Chr.) 17.

rs12946942 defined a 130-kb LD block within an approximately 2-Mb region on chromosome 17 (**Figure 1**). No RefSeq genes have been mapped in this LD block. Twenty common SNPs in the LD block were genotyped in the GWAS, the most significant of which was rs12946942 (**Figure 1**).

To further characterize the chromosome 17q24.3 locus, we imputed genotypes of additional SNPs in the locus using 1000 Genomes Project's East Asian population samples' (EAS) reference haplotypes and tested their association with AIS. SNPs rs12946942 and rs12941471 yielded the strongest evidence for association (**Figure S2A**), which were in complete LD (*r* ^2^ = 1) with each other. After conditioning on the top SNP (rs12946942), there was no secondary association signal for AIS within the region (**Figure S2B**).

## Discussion

The region defined by rs12946942 was a gene desert. The closest genes include *SOX9* and *KCNJ2* [32]. *SOX9* (MIM 608160) is a promising candidate gene for AIS as it encodes a transcription factor involved in chondrogenesis [33]. *SOX9* mutations cause campomelic dysplasia (MIM 114290), a skeletal dysplasia characterized by bowed, long bones, small scapula, tracheobronchial narrowing, sex reversal and kyphoscoliosis [34]. Very long-range cis-regulatory elements controlling tissue-specific *SOX9* expression have been previously reported [35], [36]. The LD block containing rs12946942 has recently been defined as a susceptibility locus of prostate cancer in European Caucasians [37]. The block contains six enhancer elements, of which the E1 enhancer forms a long-range chromatin loop to *SOX9* in a prostate cancer cell line. Two SNPs within the E1 enhancer were shown by in vitro reporter assays to direct allele-specific gene expression. We hypothesize that variants in this region may likewise participate in scoliosis pathogenesis by controlling scoliosis-related tissue-specific expression of *SOX9* or other genes.

*KCNJ2* (MIM 600681) is another promising candidate gene for AIS. It encodes the inward-rectifying potassium channel Kir2.1, which is a component of the inward rectifier current IK1. IK1 provides a repolarizing current during the most terminal phase of repolarization and is the primary conductance that controls the diastolic membrane potential [38]. *KCNJ2* mutations lead to a cardiodysrhythmic type of periodic paralysis known as Andersen-Tawil syndrome (ATS; MIM 170390) [39], which is characterized by ventricular arrhythmias, periodic paralysis, facial and skeletal dysmorphism including hypertelorism, small mandible, cleft palate, syndactyly, clinodactyly, and scoliosis [38], [39]. Furthermore, the 17q24.2-q24.3 micro-deletion syndrome whose deletion area includes *KCNJ2* and rs12946942 showed skeletal malformations similar to the ATS phenotype including progressive scoliosis [40]. Interestingly, a similar micro-deletion syndrome including *KCNJ2*, but not rs12946942, was not associated with a scoliosis phenotype [41].

Thus, through a Japanese GWAS followed by replication studies in Japanese and Chinese populations, we identified a susceptibility locus for severe AIS on chromosome 17q24.3 that showed genome-wide significance. This region contains a few promising candidate genes that may be associated with the disease. Further studies are now necessary to identify the causal gene and its variant in the locus.

## Supporting Information

Figure S1 **Manhattan plot showing the** ***P*** **values from genome-wide association study (minimum** ***P*** **value in allele, recessive and dominant models).** The horizontal line represents the genome-wide significance threshold (*P* = 5×10^−8^).
(TIF)

Figure S2 **Regional association plots and recombination rates of AIS susceptibility locus on chromosome 17q24.3.** The chromosome position (NCBI Build 37) of SNPs against −log_10_ [*P* value] from a logistic regression analysis is shown. (A) Unconditioned analysis. The SNP with highest association signal (rs12946942) is represented as a purple diamond. Imputed (circles) and genotyped SNPs (squares) are colored according the LD (*r* ^2^) with rs12946942. (B) Conditioned analysis. Red circles are unconditioned and gray circles are conditioned for rs12946942 (gray triangle).
(TIF)

Table S1 **Association of the 27 SNPs selected from the GWAS.**
(DOC)
