# Supplementary figures and images for: Identification of a Susceptibility Locus for Severe Adolescent Idiopathic Scoliosis on Chromosome 17q24.3
Source: PLoS One. 2013 Sep 4;8(9):e72802. doi: 10.1371/journal.pone.0072802 (PMC3762929; doi:10.1371/journal.pone.0072802)

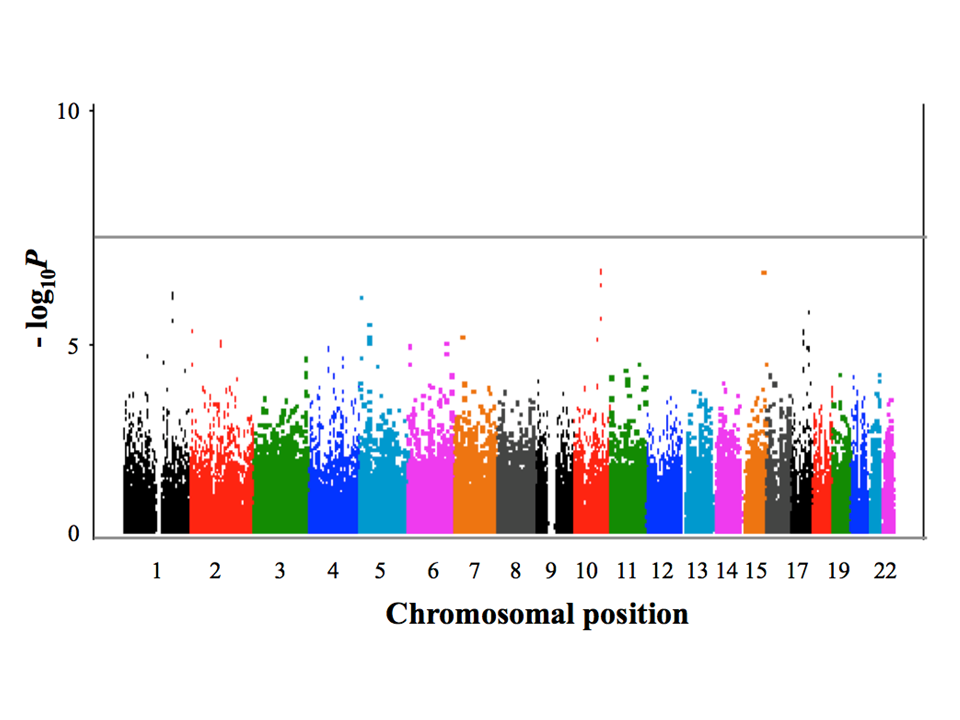

Supplement: Figure S1 — Manhattan plot showing the P values from genome-wide association study (minimum P value in allele, recessive and dominant models). The horizontal line represents the genome-wide significance threshold (P = 5×10^−8^). (TIF) [file pone.0072802.s001.tif]

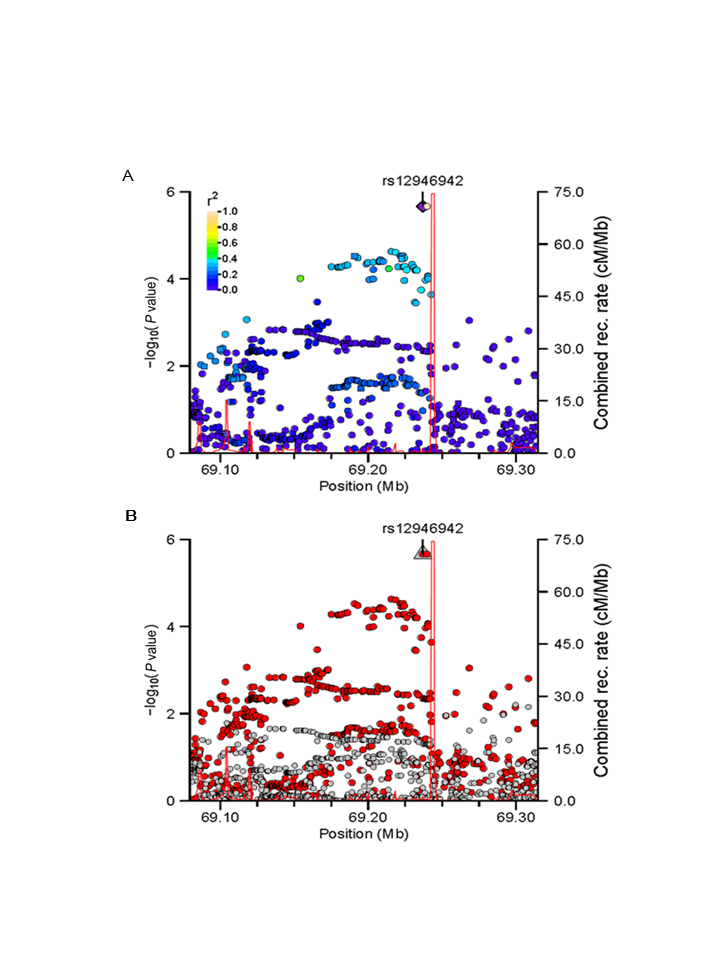

Supplement: Figure S2 — Regional association plots and recombination rates of AIS susceptibility locus on chromosome 17q24.3. The chromosome position (NCBI Build 37) of SNPs against −log10 [P value] from a logistic regression analysis is shown. (A) Unconditioned analysis. The SNP with highest association signal (rs12946942) is represented as a purple diamond. Imputed (circles) and genotyped SNPs (squares) are colored according the LD (r 2) with rs12946942. (B) Conditioned analysis. Red circles are unconditioned and gray circles are conditioned for rs12946942 (gray triangle). (TIF) [file pone.0072802.s002.tif]
