# Supplementary material for: Identification of a Susceptibility Locus for Severe Adolescent Idiopathic Scoliosis on Chromosome 17q24.3
Source: PLoS One. 2013 Sep 4;8(9):e72802. doi: 10.1371/journal.pone.0072802 (PMC3762929; doi:10.1371/journal.pone.0072802)
Supplement: Table S1 — Association of the 27 SNPs selected from the GWAS. (DOC) [file pone.0072802.s003.doc]

| **Table S1.** Association of the 27 SNPs selected from the GWAS | | | | | | | | | |
| --- | --- | --- | --- | --- | --- | --- | --- | --- | --- |
| dbSNP ID | Chromosome | RAF | | *P* value^a^ | | | Odds ratio (95 % CI) | | |
|  |  | case | control | allele | recessive | dominant | allele | recessive | dominant |
| rs11190870 | 10q24.31 | 0.661 | 0.572 | **2.72×10^-7^** | 1.93×10^-5^ | 3.10×10^-5^ | 1.46 (1.26-1.68) | 1.55 (1.27-1.89) | 1.89 (1.39-2.56) |
| rs625039 | 10q24.31 | 0.726 | 0.643 | **6.27×10^-7^** | 4.59×10^-6^ | 6.59×10^-4^ | 1.47 (1.26-1.71) | 1.58 (1.30-1.92) | 1.87 (1.30-2.69) |
| rs925203 | 5p15.31 | 0.460 | 0.375 | **7.77×10^-7^** | 7.42×10^-6^ | 2.40×10^-4^ | 1.42 (1.24-1.63) | 1.76 (1.37-2.26) | 1.48 (1.20-1.82) |
| rs7545121 | 1q31.1 | 0.612 | 0.525 | **8.33×10^-7^** | 2.50×10^-5^ | 9.31×10^-5^ | 1.42 (1.24-1.64) | 1.56 (1.27-1.92) | 1.71 (1.30-2.24) |
| rs12946942 | 17q24.3 | 0.274 | 0.203 | **1.25×10^-6^** | 1.95×10^-5^ | 1.39×10^-4^ | 1.48 (1.26-1.74) | 2.24 (1.53-3.27) | 1.47 (1.20-1.79) |
| rs11598564 | 10q24.31 | 0.539 | 0.456 | **2.43×10^-6^** | 4.74×10^-4^ | 3.02×10^-5^ | 1.39 (1.21-1.60) | 1.49 (1.19-1.86) | 1.64 (1.30-2.08) |
| rs267766 | 5p13.2 | 0.306 | 0.234 | **2.90×10^-6^** | 2.64×10^-3^ | 1.69×10^-5^ | 1.44 (1.24-1.68) | 1.74 (1.21-2.51) | 1.54 (1.26-1.87) |
| rs1367272 | 2p25.1 | 0.607 | 0.540 | 1.12×10^-4^ | **3.08×10^-6^** | 1.44×10^-1^ | 1.32 (1.15-1.52) | 1.63 (1.33-2.00) | 1.21 (0.94-1.57) |
| rs2676801 | 17q21.33 | 0.773 | 0.709 | 5.29×10^-5^ | **4.97×10^-6^** | 3.11×10^-1^ | 1.39 (1.19-1.64) | 1.59 (1.30-1.94) | 1.22 (0.83-1.79) |
| rs2047176 | 5p13.2 | 0.462 | 0.384 | **6.57×10^-6^** | 3.31×10^-5^ | 1.09×10^-3^ | 1.38 (1.20-1.58) | 1.67 (1.31-2.14) | 1.42 (1.15-1.75) |
| rs6570507 | 6q24.1 | 0.501 | 0.423 | **7.58×10^-6^** | 1.19×10^-3^ | 7.09×10^-5^ | 1.37 (1.19-1.58) | 1.47 (1.16-1.86) | 1.56 (1.25-1.95) |
| rs655540 | 11q24.2 | 0.383 | 0.314 | 3.63×10^-5^ | **8.11×10^-6^** | 7.08×10^-3^ | 1.35 (1.17-1.56) | 1.90 (1.43-2.52) | 1.31 (1.08-1.60) |
| rs9405284 | 6p25.1 | 0.741 | 0.671 | 1.91×10^-5^ | **9.48×10^-6^** | 4.49×10^-2^ | 1.40 (1.20-1.64) | 1.56 (1.28-1.90) | 1.45 (1.01-2.08) |
| rs7895098 | 10q23.1 | 0.895 | 0.841 | **1.12×10^-5^** | 8.02×10^-5^ | 2.44×10^-3^ | 1.62 (1.30-2.01) | 1.60 (1.27-2.03) | 5.13 (1.58-16.7) |
| rs9496346 | 6q24.1 | 0.509 | 0.433 | **1.36×10^-5^** | 2.19×10^-3^ | 7.67×10^-5^ | 1.36 (1.18-1.56) | 1.43 (1.14-1.81) | 1.57 (1.25-1.96) |
| rs346981 | 1p22.2 | 0.559 | 0.483 | **1.62×10^-5^** | 2.20×10^-4^ | 7.92×10^-4^ | 1.36 (1.18-1.56) | 1.50 (1.21-1.87) | 1.51 (1.18-1.92) |
| rs2852199 | 11q22.3 | 0.349 | 0.280 | **1.99×10^-5^** | 1.30×10^-3^ | 2.00×10^-4^ | 1.38 (1.19-1.60) | 1.69 (1.22-2.33) | 1.45 (1.19-1.77) |
| rs4076823 | 16p13.13 | 0.671 | 0.602 | **4.42×10^-5^** | 2.11×10^-4^ | 4.24×10^-3^ | 1.35 (1.17-1.57) | 1.45 (1.19-1.77) | 1.55 (1.15-2.11) |
| rs7101916 | 11q13.1 | 0.499 | 0.428 | **4.91×10^-5^** | 4.40×10^-4^ | 1.90×10^-3^ | 1.33 (1.16-1.53) | 1.51 (1.20-1.91) | 1.41 (1.14-1.76) |
| rs10485749 | 20p12.2 | 0.697 | 0.628 | **5.06×10^-5^** | 1.29×10^-3^ | 4.49×10^-4^ | 1.36 (1.17-1.58) | 1.38 (1.13-1.68) | 1.82 (1.30-2.56) |
| rs11227247 | 11q13.1 | 0.492 | 0.422 | **6.11×10^-5^** | 6.43×10^-4^ | 1.84×10^-3^ | 1.33 (1.16-1.52) | 1.50 (1.19-1.90) | 1.41 (1.14-1.75) |
| rs12346254 | 9p24.1 | 0.768 | 0.705 | **6.68×10^-5^** | 7.48×10^-5^ | 4.08×10^-2^ | 1.38 (1.18-1.63) | 1.49 (1.22-1.82) | 1.52 (1.02-2.26) |
| rs10485285 | 6q14.1 | 0.583 | 0.513 | **7.27×10^-5^** | 1.48×10^-4^ | 7.56×10^-3^ | 1.33 (1.15-1.52) | 1.50 (1.22-1.86) | 1.41 (1.09-1.81) |
| rs9918553 | 7p14.1 | 0.760 | 0.697 | **7.39×10^-5^** | 6.04×10^-4^ | 2.10×10^-3^ | 1.38 (1.18-1.61) | 1.41 (1.16-1.72) | 1.95 (1.27-3.01) |
| rs7143583 | 14q21.2 | 0.921 | 0.877 | **8.22×10^-5^** | 1.46×10^-4^ | 6.86×10^-2^ | 1.63 (1.27-2.08) | 1.65 (1.27-2.15) | 2.92 (0.87-9.76) |
| rs17012036 | 4q28.1 | 0.273 | 0.214 | **8.22×10^-5^** | 2.59×10^-2^ | 1.62×10^-4^ | 1.38 (1.17-1.61) | 1.59 (1.05-2.41) | 1.46 (1.20-1.78) |
| rs454578 | 5q14.1 | 0.282 | 0.224 | **9.68×10^-5^** | 7.11×10^-3^ | 4.35×10^-4^ | 1.37 (1.17-1.60) | 1.71 (1.15-2.53) | 1.42 (1.17-1.73) |
| RAF: risk allele frequency. CI: confidence interval. | | | | | | | | | |
| ^a^calculated by *x*^2^ test. The minimal *P* value among the three models for each SNP is presented in bold. | | | | | | | | | |
